# Genetic Targets and Applications of Iron Chelators for Neurodegeneration with Brain Iron Accumulation

**DOI:** 10.1021/acsbiomedchemau.3c00066

**Published:** 2024-03-11

**Authors:** Neharika Marupudi, May P. Xiong

**Affiliations:** Department of Pharmaceutical & Biomedical Sciences, College of Pharmacy, University of Georgia, Athens, Georgia 30602-2352, United States

**Keywords:** Brain iron, reactive oxidative species, PKAN, PLAN, BPAN, MPAN, iron chelation, gene therapy

## Abstract

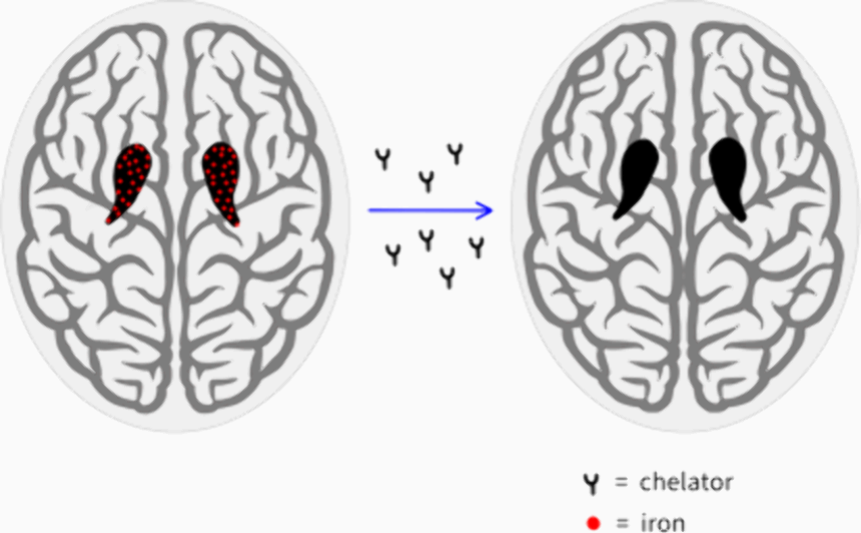

Neurodegeneration with brain iron accumulation (NBIA) is a group of neurodegenerative diseases that are typically caused by a monogenetic mutation, leading to development of disordered movement symptoms such as dystonia, hyperreflexia, etc. Brain iron accumulation can be diagnosed through MRI imaging and is hypothesized to be the cause of oxidative stress, leading to the degeneration of brain tissue. There are four main types of NBIA: pantothenate kinase-associated neurodegeneration (PKAN), PLA2G6-associated neurodegeneration (PLAN), mitochondrial membrane protein-associated neurodegeneration (MKAN), and beta-propeller protein-associated neurodegeneration (BPAN). There are no causative therapies for these diseases, but iron chelators have been shown to have potential toward treating NBIA. Three chelators are investigated in this Review: deferoxamine (DFO), desferasirox (DFS), and deferiprone (DFP). DFO has been investigated to treat neurodegenerative diseases such as Alzheimer’s disease (AD) and Parkinson’s disease (PD); however, dose-related toxicity in these studies, as well as in PKAN studies, have shown that the drug still requires more development before it can be applied toward NBIA cases. Iron chelation therapies other than the ones currently in clinical use have not yet reached clinical studies, but they may possess characteristics that would allow them to access the brain in ways that current chelators cannot. Intranasal formulations are an attractive dosage form to study for chelation therapy, as this method of delivery can bypass the blood-brain barrier and access the CNS. Gene therapy differs from iron chelation therapy as it is a causal treatment of the disease, whereas iron chelators only target the disease progression of NBIA. Because the pathophysiology of NBIA diseases is still unclear, future courses of action should be focused on causative treatment; however, iron chelation therapy is the current best course of action.

## Introduction

Neurodegeneration with brain iron accumulation (NBIA) is an overall term to describe a group of rare neurodegenerative diseases that are inherited through a variety of genes that result in the presentation of iron overload in the brain. NBIA is characterized by extrapyramidal movement disorders and abnormal iron accumulation in the deep basal ganglia nuclei.^1^ The cumulative term for NBIA diseases comes from the genes that are responsible for the iron overload in the brain, but the exact mechanism for this manner of neurodegeneration is not completely understood.^2^ Nomenclature for NBIA diseases has generally been regulated with the “(mutant protein)-associated neurodegeneration” convention.^1^ NBIA can be classified as an orphan disease, affecting 1–3/1,000,000 in a population, with autosomal dominant, autosomal recessive, or X-linked dominant inheritance.^3^ Treatments for NBIA that have been developed have been symptomatic in nature; there are no causal therapies targeting iron accumulation. Iron chelation therapy is a potential symptomatic therapy. Iron chelating agents have been proven to induce iron excretion and a negative iron balance in patients with thalassemia major and hemochromatosis.^4^ These therapies have potential to be used in cases of NBIA and have been used with some benefits to mitigate brain iron accumulation. In this paper, we will review the mechanisms of NBIA and the role of iron in neurological function, the main genetic targets of NBIA and the use of iron chelators to treat this disease, and whether iron chelation is a viable therapy option to treat these diseases. We will also discuss some other potential new therapies that can be applied as well.

## Mechanisms of NBIA

Metals such as iron, zinc, and copper are vital to the function of multiple biological processes in the brain including nerve transmission, the synthesis and metabolism of neurotransmitters, and oxygen transport.^5^ Iron is present in dopaminergic neurons, where it is vital in dopamine synthesis and electron transport chain production.^4^

## Mechanism of Iron Toxicity

Most iron in the body is related to the heme moiety of hemoglobin and myoglobin transport proteins.^6^ Iron plays a vital role in oxygen transport through the blood, in electron transport within cells, and as a cofactor for various enzymes in the body.^6^ However, an overabundance of this metal can lead to extensive damage of cells and organs. Oxidative stress is a deviation from the steady state redox balance of the body’s metabolic system, or redox homeostasis.^7^ Transition metals such as iron can induce oxidative stress by reacting with hydrogen peroxide to produce hydroxyl radicals in a process known as the Fenton reaction, seen below.^8^
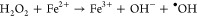
The Fenton reaction plays a role in a larger mechanism, the Haber-Weiss reaction, in which iron plays the role of catalyst; as the reaction is thermodynamically unfavorable in biological systems, the presence of iron is what allows for the reaction to proceed.^8^ Below are the steps of the Haber Weiss reaction.
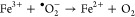

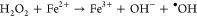
Net equation:
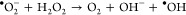
The reaction indicates a direct relationship between the redox-active iron and the hydroxyl radical, which is incredibly reactive and therefore very dangerous.^9^ The hydroxyl ion is an example of a reactive oxygen species (ROS) that is produced by iron, which can damage lipids and proteins.^10^ Iron accumulation leading to an increase in the labile iron pool (LIP) is therefore known to be associated with tissue damage due to oxidative stress.^11^ The LIP is characterized as a deposit of excess chelatable and redox-active iron in both its ferric and ferrous forms and could potentially participate in redox cycling, which refers to the process of iron being reduced and oxidized to form the hydroxyl radical.^12,13^ Excess iron that enters the LIP can react with the byproducts of cellular respiration, such as hydrogen peroxide, to produce ROS that are responsible for cellular damage.^14^ Some examples of the ways in which ROS can damage a cell are by inducing apoptosis, autophagia, necroptosis, and ferroptosis.^15^

Another detrimental consequence of the production of ROS is lipid peroxidation. The reaction takes place in three steps: initiation, propagation, and termination. The radical ROS initiates the reaction by reacting with a polyunsaturated fatty acid (PUFA) to create a lipid radical, which then reacts with molecular oxygen to form a reactive lipid peroxide radical LOO^*•*^ and terminates following a hydrogen transfer from a neighboring lipid to create LOOH, a lipid peroxide.^16^ Lipid peroxidation alters physical properties of lipid bilayers, thus affecting membrane assembly, structure, and dynamics, and lipid peroxide radicals can further propagate by contributing to the production of variety of ROS.^17^ Due to its high lipid content, the brain is most susceptible to peroxidative damage, which directly correlates to regional iron concentration.^18^

### Homeostasis of Iron in the Body

Regulation of iron in the body is important because there is no efficient physiological pathway to excrete iron from the body.^19^ Ferritin, an iron-storage protein found in cells throughout the body, is a key factor in iron homeostasis due to its capability to store excess iron and release it as needed.^20^ Dietary iron enters the body and is transported in two forms: heme iron and nonheme iron. Heme iron is present in hemoproteins such as myoglobin (stored in muscle cells) or hemoglobin (stored in red blood cells), whereas nonheme iron is directly transported in the blood by transferrin.^21^ The absorption of dietary iron can only occur in the form of heme iron or when the iron atom is in the ferrous (Fe^2+^) state. More specifically, nonheme iron must be transformed from its insoluble ferric (Fe ^3+^) form into ferrous iron by specialized enzymes on the brush border of enterocytes before it can be transported across membranes by an iron export protein known as ferroportin 1 (FPN1).^22^ An important transport protein present in the intestinal epithelium is the divalent metal transporter 1 (DMT1), which transports ferrous iron across the apical membrane of the brush border, whereas FPN1 is located on the basolateral membrane of the cell.^23^ It is these epithelial enterocytes and their transmembrane proteins transporters that line the villi in the gastroduodenal junction that are responsible for controlling heme and nonheme iron absorption in the body, where it then passes through the gut lumen into the plasma.^19^

Even after it has been absorbed, iron transport must be strictly managed by a multitude of molecules to maintain homeostasis. Iron regulation in the body is controlled by hepcidin, a peptide produced in the liver, which binds to FPN1 to be internalized and degraded, inhibiting the excessive release of iron into the blood circulation.^24^ Transferrin, a liver protein able to bind two ferric ions, can safely circulate regulated iron to most cells in the body, including neurons, without causing any ROS production.^24^ This process is facilitated by hephaestin, a membrane-bound ferroxidase (iron oxidizing enzyme), which oxidizes Fe^2+^ back to Fe^3+^ prior to transport by transferrin.^25^ Transferrin binds to the transferrin receptors TfR1 and TfR2, which have slightly different cellular uptake pathways that result in varied phenotypic presentations in cells: TfR1 downregulation results in low iron levels in the tissues, while TfR2 downregulation can result in hemochromatosis.^26^ Due to the focus of this review being the brain, the reader is directed to more detailed review papers on mechanisms of iron regulation and metabolism.^27−29^

### Iron Regulation in the Brain

The brain is different from other organs in the body because of the vascular barrier that it lies behind, which regulates the physical material exchange between blood and fluids and the brain tissue; because of this, iron cannot be directly absorbed into the brain from systemic circulation.^30^ Iron uptake into the brain is regulated by the blood-brain barrier (BBB), made up of cerebrovascular endothelial cells, that controls the passage of large molecules across the membrane in a multistep transcellular process.^30^ Systemic circulation is separated by the BBB from the central nervous system (CNS), which does have the branching capillaries of systemic circulation that allow for passage of drugs and nutrients.^31^ Studies on oligodendrocytes, glial cells of the CNS that produce myelin, found that iron is integral for their function.^32^ Traces of ferritin, transferrin and iron were found in these cells, and a direct correlation between iron acquisition and myelin production was discovered.^33^

The choroid plexus is a network of capillaries in the brain that has many functions, with one of them being the production of cerebrospinal fluid (CSF) and separating the brain from the bloodstream.^34^ The capillaries of the choroid plexus extend into the four ventricles of the brain and regulate the drugs and nutrients that pass through.^31^ A wide range of transporters are expressed in the choroid plexus, such as Menke’s and Wilson’s metal transporters and DMT1, that could play a role in iron regulation in the brain along with the BBB.^35^

After iron attached to holo-transferrin, or bound-transferrin, enters the brain, whether is it through the choroid plexus or the BBB, it travels into the ventricles via interstitial fluid or cerebrospinal fluid (CSF) and binds to surface cellular transferrin receptor 1 (TFRC); the holo-transferrin is then endocytosed into endothelial cells in the CNS.^25^ Astrocyte end-feet that sheath the endothelial cells play an important role in the maintenance of the BBB and the regulation of iron transport across the membrane by regulating the hydrogen ion concentration, which is directly correlated with the release of iron in the endothelial cells.^36^ The clearance of iron from the CNS is debated, but one potential pathway is through CSF drainage; iron efflux may be related to the iron concentration gradient in the CSF and interstitial fluid.^37^

## Genetic Targets

### Pantothenate Kinase-Associated Neurodegeneration (PKAN)

There are many various types of NBIA that can be differentiated by the mutated genes that cause the disorder as well as magnetic resonance imaging (MRI). The most prevalent form of NBIA, pantothenate kinase-associated neurodegeneration (PKAN), also known as NBIA1, makes up ∼35–50% of the NBIA patient population.^38^ PKAN is caused by the gene PANK2 located on chromosome 20p12.3 and presents in two variations: typical and atypical PKAN.^39^ PANK2 is an autosomal recessive gene that causes an error in the phosphorylation of Vitamin B_5_, also known as pantothenate, which is a micronutrient vital in the production of coenzyme A (CoA) in the mitochondria.^40^ Pantothenate kinase is the enzyme that catalyzed the reaction of pantothenate into CoA, allowing it to directly affect cellular energy metabolism.^41^ The downstream effect that the PANK2 mutation has on the biosynthesis of CoA, affecting the rate-limiting step of the pathway, is associated with decreased acetylation of histones and tubulin, which is implicated in the impairment in neuronal functional that can lead to neurodegeneration.^42^ The impaired neurons have decreased respiratory ability in the mitochondria, increasing the levels of ROS in PKAN patients compared to baseline levels, which directly links oxidative stress to the PANK2 deficiency mutation.^43^ Typical PKAN is early onset with a rapid progression of symptoms such as dystonia, dysarthria, spasticity, hyperreflexia, and extensor toe signs; atypical PKAN presents with a later onset and slower progression of the typical symptoms, with speech difficulty and psychiatric symptoms also developing.^44^ PKAN is differentiated from other forms of NBIA by its specific “eye of the tiger sign” on radiographic imagery, consisting of a hypointensity of the global pallidus, hypointensity of the substantia nigra, and dentate nucleus.^45^

The globus pallidus is part of the basal ganglia, masses of neuronal cell bodies located within the cerebrum. Also known as the subcortical nuclei, the basal ganglia control voluntary functions. The globus pallidus is the medial portion of the lentiform nucleus, the lower mass of the most prominent basal ganglia, known as the corpus striatum. The substantia nigra is another nucleus in the brain and is the primary source of input to the basal ganglia via its dopaminergic neural projections.^46^ The nigrostriatal pathway is heavily implicated in the pathology of the motor deficits that are present in NBIA diseases.^46^ The dentate nucleus is the largest deep cerebellar nucleus and is involved in the efferent modulation of motor neurons; disruption of dentate nucleus function is associated with cerebellar ataxia.^47^

### PLA2G6-Associated Neurodegeneration (PLAN)

PLA2G6-associated neurodegeneration (PLAN) is aptly named for the autosomal recessive PLA2G6 mutation that causes the disorder. The most common type of this disorder is infantile neuroaxonal dystrophy (INAD) presents with severe psychomotor symptoms such as rapidly progressing hypotonia, hyperreflexia, and tetra paresis and cerebellar atrophy with brain iron accumulation.^48^ The PLA2G6 gene is vital in cellular membrane homeostasis as it encodes for the iPLA_2_-Via protein, a calcium-independent phospholipase, which has an underlying connection to the axonal pathology of PLAN, indicating that neuron axons are damaged when the mutation is expressed.^49^ Neuropathology studies indicate that the PLA2G6 mutation results in axonal spheroids in the cerebral cortex, striatum, cerebellum, brainstem and spinal cord.^50^ Axonal spheroids are bead-like swellings along axons and are a frequent characteristic of axonal degeneration.^51^ Lewy bodies (LBs) are commonly found in many neurodegenerative diseases and are made up of aggregated forms of the protein alpha-synuclein (α-Syn), which form beta-sheet-rich amyloid fibrils that contribute to the pathology of the diseases.^52^ LBs are reportedly found in cases of PLAN and were confirmed through genetic testing, indicating that the PLA2G6 mutation potentially induces the aggregation of α-Syn.^52^ PLAN shared similar pathological characteristics with Parkinson’s disease (PD) and Alzheimer’s disease (AD), which could be caused by the Lewy bodies present in the neurological diseases.^48^ Both PKAN and PLAN involve a mutation associated with the mitochondria and lipid metabolism, which in turn could alter the regulation of iron transport and utilization in the brain.^48^ Though the exact pathology of the forms of NBIA are not yet fully understood, it can be inferred that this pathway plays an instrumental role.

### Mitochondrial Membrane Protein-Associated Neurodegeneration (MPAN)

The next most prevalent form of NBIA is mitochondrial membrane protein-associated neurodegeneration (MPAN), accounting for ∼6–10% of all NBIA cases.^38^ Caused by the autosomal recessive gene C19orf12, MPAN usually presents in childhood or adolescence with symptoms such as dystonia-parkinsonism, optic atrophy, and axonal motor neuropathy.^53^ T2-weighted MRI displays hypo-intensities indicative of iron accumulation in the globus pallidus interna and externa and iso-intensities in the medial medullary lamina.^53^ Compared to PKAN, MPAN has a later onset and a more gradual psychomotor regression; the characteristic “eye-of-the-tiger” sign on MRI scans is not present, whereas a prominent T2 hypointensity in the substantia nigra is present.^54^ Lewy bodies and neurites are widespread in the globus pallidus, corpus striatum, midbrain substantia nigra, neocortex, and hippocampus and are present in a substantially higher volume than in cases of sporadic Lewy body disease.^55^ The C19orf12 gene is located on chromosome 19q12 and encodes for the C19orf12 protein.^56^ The function of this protein is not clear, but the expression of the gene in fat cells and its coregulation with other genes involved in fatty acid metabolism implicate its role in lipid metabolism.^57^

### Beta-Propeller Protein-Associated Neurodegeneration (BPAN)

The last of the four most common types of NBIA is beta-propeller protein-associated neurodegeneration (BPAN). The gene responsible for this disorder is WDR45, which lies on the X chromosome, making BPAN the only NBIA disorder caused by an X-linked dominant gene.^58^ BPAN is the most recently discovered form of NBIA, with the de novo WDR45 mutation leading to iron accumulation in the substantia nigra and globus pallidus, along with axonal spheroids, gliosis, severe neuronal loss, and a significant decrease in Purkinje cells.^59^ The WDR45 gene encodes a protein belonging to the LIS1 family, all important for neuronal survival, and can be characterized as a beta-propeller scaffold, referring to the tertiary structure of the protein, that allows for protein–protein interactions that are critical in the process of autophagy.^60^

Autophagy is the degradation of cellular components that is activated because of low nutrient availability to dispose of organelles, misfolded/aggregated proteins, or infectious agents.^61,62^ As mentioned before, protein aggregation resulting in cellular protein degradation is linked to the mechanism of NBIA. While much is not yet understood about the pathogenesis of BPAN, a mutation causing a defect in the pathways of autophagy could certainly be implicated in playing an instrumental role in the development of iron accumulation due to the iron homeostasis pathways mentioned previously. Axial T-2 imaging indicates hypointensity in the globus pallidus consistent with iron accumulation, cortical atrophy, and even more obvious hypointensity in the substantia nigra.^63^ Axial T-1 imaging show a combination of hypointensity in the mesencephalic peduncles with a linear hyperintensity surrounding it, often referred to as a “halo sign” typical of BPAN and may be unique to this disorder.^63^

Table 1 summarizes each subtype of NBIA with the monogenetic mutation, inheritance pattern, prevalence, and therapeutics available or in development.

**Table 1:** Currently Known Subtypes of NBIA Categorized by Gene, Percentage of Total NBIA Cases, and Current Therapies and Their Stages of Development

Type of NBIA	Gene	Inheritance	Approximate percentage of total NBIA cases	Therapeutics available and stage of development	Results
Pantothenate kinase-associated neurodegeneration (PKAN)	PANK2	Autosomal recessive	50%	Deferiprone (phase II–III)	Significant reduction in brain iron, increased improvement of neurological symptoms, and increased quality of life
Phospholipase A2-associated neurodegeneration (PLAN)	PLA2G6	Autosomal recessive	20%	RT001 (phase II/III)^64^	Active, not recruiting^64^
Mitochondrial membrane protein-associated neurodegeneration (MPAN)	C19orf12	Autosomal recessive	10%	None	n/a
Beta-propeller protein-associated neurodegeneration (BPAN)	WDR45	X-linked dominant	7%	None	n/a
Fatty acid hydroxylase-associated neurodegeneration (FAHN)	FA2H	Autosomal recessive	1%	None	n/a
Coenzyme A synthase protein-associated neurodegeneration (CoPAN)	COASY	Autosomal recessive	2%	None	n/a
Kufor-Rakeb syndrome	ATP13A2	Autosomal recessive	2%	None	n/a
Woodhouse-Sakati syndrome	DCAF17	Autosomal recessive	2%	None	n/a
Neuroferritinopathy	FTL	Autosomal dominant	2%	None	n/a
Aceruloplasminemia	CP	Autosomal recessive	2%	Deferiprone (early phase I)^65^	No results posted^65^

## Iron Chelation Therapy

After identifying the most common types of NBIA and the mechanisms behind those specific diseases, the possible avenues of treatment can now be explored. Chelators are substances that can form complexes with metal ions. Iron chelation therapy is a promising treatment for the disease, but it is still symptomatic and not causal, or addressing the root of the illness. Iron chelators have a high affinity to bind to iron but must also be able to cross or bypass the blood-brain barrier to access the excess iron without causing significant iron depletion.^66^ The three iron chelators currently available for clinical use are deferoxamine (DFO), desferasirox (DFS), and deferiprone (DFP).

DFO is the oldest and the most widely used iron chelator currently on the market. Available as a subcutaneous infusion because of its short half-life in humans, DFO has had a consistently beneficial effect on the long-term survival of patients with thalassemia.^67^ It is a hexadentate chelator and binds to iron in a 1:1 molar ratio to be excreted through the urine or bile.^68^ The pharmacokinetic and biodistribution properties of the drug were improved using thioketal-cross-linked polymeric nanogels postfunctionalized with DFO moieties (rNG-DFO), yielding good elimination and low cytotoxicity profiles while also reducing iron-mediated oxidative stress.^69^

While is it considered to be a safe and effective treatment for transfusional iron overload, low patient compliance and poor pharmacokinetics of the free drug administered parentally hinders its use and effectiveness in patients. Furthermore, DFO has a low lipophilicity and a large molecular weight, barring it from being able to cross the BBB to treat NBIA.^70^ Oral dosage forms of iron chelators are of interest for their increased patient compliance but DFO has been reported to have poor oral bioavailability. Thus, oral formulations that are reported are prophylactic in nature and focus on chelating dietary iron in the gut to reduce overall dietary iron uptake in patients with nontransfusional hemochromatosis.^71^ The parenteral route of delivering various types of nanoformulations of DFO to reduce body iron stores in transfusional hemochromatosis conditions remains the most popular, yet few of these studies have focused on designing IV nanoformulations that can efficiently cross the BBB.

Overall, the intranasal (IN) route seems to be the most favorable for bypassing the BBB and treating a multitude of neurodegenerative diseases with DFO. For example, IN-DFO was shown to target the CNS to reduce the infarct volume of middle cerebral artery occlusion and significantly improve spatial memory in mouse models with AD.^72,73^ Chronic IN-DFO was used in an α-syn rAAV vector-based model of PD and showed an improvement in motor performance and a reduction in the number and size of α-syn aggregates.^74^ DFO can be rapidly delivered to the brain through this route along the olfactory and trigeminal nerves with negligible systemic adverse side effects.^75^ Intranasal formulations have been preferred in many cases over parenteral and oral formulations, but there are concerns regarding limitations of IN efficiency due to the protective barriers of the nasal mucosa, which result in the necessity for both frequent and high doses of IN formulations, leading to irritation of the nasal mucosa.^76^

DFP was the first oral chelator that proceeded to enter extensive human trials.^67^ It was shown to have short-term safe and effective iron chelation abilities.^77^ It is a bidentate chelator, binding in a 2:1 chelator/iron ratio that is also excreted through the urine along with the free drug.^78^ DFS is tridentate oral iron chelator used to treat thalassemia and binds to iron in a 3:1 molar ratio.^79^ Clinical studies comparing the therapeutic efficiency and the tolerability of DFS to DFO report that DFS was noninferior to DFO and produced higher satisfaction and compliance.^79^ DFP is a hydroxypyridinone (HPO)-based chelator, a class of compounds that have a high selectivity for iron and a scaffold that is capable of a variety of biological actions.^80^ HPOs can be made more lipophilic by N-alkylation so that they are capable of crossing the BBB, which makes them promising candidates for treating NBIA.^81^ The downside to DFP is that it is rapidly metabolized in the liver, and there are dose-related toxicity concerns due to side effects such as agranulocytosis and mild neutropenia.^70^ DFS faces similar concerns, but its toxicity is much more severe, as there are reported fatalities associated with long-term use of the drug.^70^

Out of these three chelating agents, the applications of DFP and DFS in NBIA have been studied due to their oral formulation.^82^ DFP has been used in multiple clinical trials to treat PKAN. It was first used to treat NBIA in 2008 in a patient who had severe gait impairment and choreic dyskinesias incidence, both of which was improved significantly by treatment with DFP.^83^ A phase II pilot trial treated PKAN patients with DFP for 6 months and observed a significant reduction in iron levels in the globus pallidus, revealed by MRI scans.^84^ Another trial observed the long-term use of DFP to reduce brain iron overload and improve neurological manifestations; the 4-year follow up confirmed the safety of the drug and the efficacy of DFP as a therapeutic option in 83% of adult patients at early stages of the disease.^85^ One specific patient case study reported that a daily treatment of DFP combined with baclofen in a patient with classic PKAN decreased dystonia and increased quality of life.^86^ The TIRCON2012 V1 trial was an 18-month long, randomized, double-blind, placebo-controlled study conducted in hospitals in Germany, Italy, England, and the United States.^87^ Patients with PKAN were dosed with oral DFP twice a day, and results suggested slowed disease progression, though not significantly enough to be conclusive.^87^ An extension of the study was conducted, TIRCON-EXT (Table 2). This trial allowed for integral information to be collected on determining end points and understanding the natural history of the disease that has been used to design future clinical trials to study DFP to treat NBIA. No evidence was found of this trial progressing to the next phase of clinical trial, which is a cause for concern and leads to questions regarding how safe and effective the deferiprone treatment was.

**Table 2:** Clinical Trials Studying DFP as the Intervention for PKAN

Study	Intervention	Phase	Trial length/status	Enrollment	Dosage
Long-term Deferiprone Treatment in Patients With Pantothenate Kinase-Associated Neurodegeneration (TIRCON-EXT)^88^	Deferiprone oral solution	Phase III	June 26, 2014–March 16, 2018; completed	68	up to 15 mg/kg of body weight, twice a day
Efficacy and Safety Study of Deferiprone in Patients With Pantothenate Kinase-associated Neurodegeneration (PKAN)^89^	Deferiprone oral solution	Phase III	December 13, 2012–January 11, 2017; completed	89	80 mg/mL oral solution will be administered twice daily (b.i.d.) for 18 months. An initial dose of 5 mg/kg b.i.d. will be administered for 6 weeks. The dose will then be escalated to 10 mg/kg b.i.d. and finally to 15 mg/kg b.i.d.
Compassionate Use of Deferiprone in Patients With PKAN^90^	Deferiprone		December 21, 2015–; no longer available		Ferriprox (deferiprone) 100 mg/mL oral solution

Due to it making up the largest percentage of NBIA patients, PKAN studies are most prevalent in the literature. However, there have been studies done to assess different therapeutic options for other forms of NBIA. For example, docosahexaenoic acid (DHA) is integral in the function of the calcium-independent phospholipase A_2_β (iPLA_2_β) that is implicated in the pathology of PLAN.^91^ iPLA_2_β selectively hydrolyzes DHA, but when the protein is defective, as it is in cases of PLAN, the metabolism and signaling pathways of DHA in the brain are significantly reduced, increasing its vulnerability to neuroinflammation.^91^ Another study reported decreases in not only DHA metabolism in iPLA_2_β-deficient mice but also altered expression and concentrations of other brain phospholipases and fatty acids, which highlights how important iPLA2β is in brain lipid metabolism and how any disturbances can cause neuropathological abnormalities.^92^ DHA has been proven to have an antioxidant effect in the brain, reducing the production of ROS and oxidative stress.^93^ DHA has been combined with metal chelation therapy (iron and copper) to treat colorectal cancer cells, specifically targeting ROS that induces cancer cell toxicity and triggers degradation.^94^ A combined therapy of DHA with an EGCG-derivative showed an improvement in the antioxidant capacities of EGCG, which can be applied to developing treatments for neurodegenerative diseases.^95^

## Novel Iron Chelation Therapies

Along with the current small molecule iron chelating agents that are being studied for their applications toward NBIA, there is a multitude of promising multifunctional agents that can also be applied to these diseases (Table 3). Epigallocatechin-3-gallate (EGCG), a polyphenol derived from tea and also known as a catechin, possesses ROS-scavenging capabilities as well as an affinity for binding and chelating metals such as copper and iron.^96^ Green tea polyphenols such as EGCG may be involved in the regulation of antioxidant protective enzymes as well; many preclinical animal studies have shown EGCG to have increased levels of antioxidant enzymes and to inhibit protein aggregation.^97^*R*-Apomorphine (*R*-APO) is a dopamine D_1_-D_2_ receptor agonist that has been successfully used to treat PD and has been proven to have neuroprotective properties.^98^ Apomorphine’s neuroprotective properties can be attributed to its antioxidant and free radical scavenging characteristics.^99^ Apomorphine also has metal chelating abilities for iron and copper ions.^100^ Benzylisoquinolines other than apomorphine have been shown to reduce Fe^3+^ and inhibit the hydroxyl radical production in the Fenton reaction, which can be applied to treatment the pathology of many neurodegenerative diseases caused by iron overload.^101^ Curcumin (CURC), capsaicin (CAP), and *S*-allylcysteine (SAC), which are active agents in spices such as turmeric, chili, and garlic, can bind to ferrous ions (Fe^2+^) to reduce lipid peroxidation by decreasing the amount of available iron to the Fenton reaction.^102^ Two newly developed iron chelators, VK-28 and M30, were studied against lactacystin-induced nigrostriatal degeneration and were found to be able to chelate iron successfully at the same potency as DFO, but unlike DFO, these chelators can cross the blood-brain barrier, which opens them up to a variety of possible clinical uses.^103^ VK-28 and M30 are derivatives of 8-hydroxyquinolines, a family of compounds that can form complexes with divalent metal cations such as iron.^104^ Clioquinol is a member of this family that is primarily used as an antibiotic and to treat pulmonary fibrosis, but it has also been used in cases of AD/PD with success.^4,105−107^ These studies have made clioquinol an attractive compound for neurodegeneration treatments, but clioquinol has also been proven to cause cytotoxicity in astrocyte-derived KT-5 cells by depleting ATP levels and increasing ROS in the cells.^108^ Though clioquinol may not be a viable therapy for NBIA, related compounds such as VK-28 and M30 have the same chelation properties without the cytotoxicity of their relative, so their application toward these diseases has potential.

**Table 3:** Novel Iron Chelation Therapies with Potential Applications toward NBIA

Intervention	Properties/characteristics	Diseases that are being studied for efficacy
Epigallocatechin-3-gallate (EGCG)^98,109^	Antioxidant effects; protein aggregation inhibition; molecular target inhibition; brain permeable	Cervical and prostate cancer
*R*-Apomorphine (*R*-APO)^98,99,110^	Dopaminergic activator; antioxidant effects and free radical scavenger	Parkinson’s disease
Benzylisoquinolines^101,111^	Deoxyribose degradation inhibitor; lipid peroxidation inhibitor; free radical scavenger	Alzheimer’s disease
Curcumin (CURC)^102,112,113^	Antioxidant, anti-inflammatory, antitumor, and antibacterial effects; upregulator of antioxidant proteins	Hypoxia-induced myocardial infarction injury and toxic chemical induced liver injury
Capsaicin (CAP)^102,114^	Analgesic, antioxidant, and anti-inflammatory effects; tumor growth inhibition; lipid oxidation inhibitor; free radical scavenger	Arthritis, diabetic neuropathy, gastric lesions, and cardiac excitability
S-allylcysteine (SAC)^9,115−117^	ROS scavenger; enzymatic and nonenzymatic antioxidant activator; prooxidant enzyme and lipid peroxidation inhibitor	Hepatocellular carcinoma and brain ischemia
VK-28^103,118^	Brain permeable; peroxidation inhibitor; iron chelator	Lactacystin-induced nigrostriatal degeneration and Parkinson’s disease
M30^92,119^	Iron chelator; free radical scavenger; brain permeable; dopamine, serotonin, and noradrenaline activator	Lactacystin-induced nigrostriatal degeneration and Parkinson’s disease

## Potentials of Gene Therapy

While iron chelators show much promise toward the treatment of NBIA, the drugs only target the iron accumulation in the brain and are not able to address the mutation that causes the genetic disorder. Gene therapy is a precision treatment for these monogenic disorders because it would correct the genetic mutation at the root of the neurodegeneration.^120^ This would be a causal treatment, where the current treatments are symptomatic. New technological developments in antisense oligonucleotide (ASO) therapy, adeno-associated virus (AAV) vector gene delivery, and CRISPR-Cas9 genome editing could be used as a clinical treatment for NBIA.^120^

ASO therapy utilizes oligonucleotides, approximately 10–30 nucleotides long, that are capable of binding to cellular RNA and affecting multiple RNA processes, such as splicing, transcription, and more; while they are currently unable to cross the BBB, ASOs can be injected into CSF to experience neuronal uptake into the brain.^121^ These characteristics of ASOs give the molecules a high target specificity and allow them to be applied as treatment for undruggable diseases.^122^ Amyotrophic lateral sclerosis (ALS) and other neurodegenerative diseases are being tested with ASO treatments and have determined that ASOs are a strong candidate for monogenetic disorder therapies.^123^ Adeno-associated virus (AAV) vectors are another possible gene therapy that has shown promise in treating diseases like NBIA. Recombinant AAVs (rAAVs) have all viral encoding removed, except for protein coding sequences, and are instead encoded with “therapeutic gene expression cassettes,” which are then released into the nucleus to be expressed in the host cells.^124^ AAV vectors have a low cytotoxicity and immunogenicity in vivo, can cross the BBB, and produce lifelong transgene expression, making them viable treatment candidates for NBIA.^124,125^ AAV vectors have been studied in PD clinical trials with promising results.^109^ Lastly, clustered regularly interspaced short palindromic repeat (CRISPR)-Cas-associated nucleases are an avenue of gene therapy that have the potential to introduce heritable genome changes; the Cas9 protein, specifically, has been proven to be reliable and robust in function, making it a suitably programmable tool to generate DNA.^126^ AAVs have been used as a delivery system for CRISPR genome editing, along with Cas9 ribonucleoprotein, due to their versatility and adaptability to many different types of host cells.^126^ CRISPR-Cas9 genome editing has been used to treat AD, PD, ALS and other neurodegenerative diseases and were shown to be successful in either reducing or reversing the mutation expression of disease pathologies.^127^ Challenges that arise with this form of therapy lie in target delivery, and current animal models do not illustrate the exact motor or cognitive features of the human disease.^120^ However, with future studies in larger animals with more similar phenotypes for NBIA, there is potential in gene therapy to be used in the future to causally treat NBIA.

## Discussion/Conclusion

Though several novel small molecule iron chelation therapies have not yet reached clinical trials to be tested in humans with neurodegenerative diseases, their applications toward NBIA provide many possible treatment options in the future. The genes that cause NBIA do not have many similarities in location or function, but the result of the mutations are associated with membrane or mitochondrial protein malfunction, leading to the accumulation of iron and harmful ROS in the brain. Enough is known about the mechanisms of NBIA to affirm that iron accumulation plays a pivotal role through the production of ROS, which leads to oxidative stress and lipid peroxidation in the brain, thus causing tissue damage and neurodegeneration. Lipid metabolism and the integrity of the phospholipid membrane are equally as important in metabolic pathways in neural cells, as well as other neuropathological features mentioned in this paper, such as Lewy bodies and altered mitochondrial function.^128^ These characteristics seem to be unifying theories throughout the different types of NBIA. Understanding how they all link together in the pathophysiology of neurodegenerative diseases caused by iron accumulation is the key to treating the cause of the disease, not only its progression.

DFP has been used in the treatment of PKAN with promising results in clinical trials. Its use in other forms of NBIA is a possibility for future studies, as well as other iron chelators once they have reached clinical studies. However, these trials have not progressed in the last five years, which raises questions whether the adverse side effects in patients are worth the chelating ability of the drugs. Because NBIA diseases are orphan diseases, there is not much motivation in funding larger clinical trials to further assess chelating treatments. There is no standard course of treatment for NBIA, and the only drugs that are approved for use are to alleviate the neuromuscular symptoms of iron overload. For example, baclofen is used to treat muscle spasms and cannot target the iron accumulation directly. However, new discoveries and developments in iron chelation therapies used to treat illnesses such as AD and PD may be applied toward NBIA, since iron overload is characteristic in many neurodegenerative diseases and its presence is positively correlated with increased oxidative damage. For example, the applications of DFO to AD and PD was reported in preclinical studies to decrease oxidative stress and improve behavior.^129^ IN-DFO has been studied in AD and PD models with encouraging results; therefore, intranasal formulations of DFP and DFS could also be an alternate route of delivery to the CNS while diminishing the chances for adverse systemic side effects. DFP has been studied to treat diseases similar to NBIA, most recently in a phase II trial to treat PD. Results showed that treatment with deferiprone decreased nigrostriatal iron content, but DFP was also associated with worsened symptoms of parkinsonism when compared to the placebo and patients presented with serious adverse events such as agranulocytosis and neutropenia.^130^

The largest challenge that iron chelators present when being used as treatment for NBIA is dose-related toxicities in the brain. Both DFP and DFS have been reported to have side effects that range from mild to fatal. A pharmacokinetic and safety profile of DFP in patients with sickle cell disease indicated that the drug was well tolerated with no major safety concerns.^131^ Studies of DFP in brain iron overload have also proven that the drug is well tolerated in the body but also produce some severe adverse effects, mentioned previously.^132^ The dose of DFP used in the phase II PD trial was lower than those used to treat systemic iron overload (30 mg/kg vs 100 mg/kg for transfusion-dependent thalassemia), but similar adverse effects were seen.^130^ Further research should be conducted to gain a deeper understanding of the toxicity of iron chelation therapy in the brain, as the results are unclear with comparison to systemic toxicity.

Iron chelators that have been tested in clinical trials have been successful in reducing iron content in the brain, but the adverse events that occur along with treatment are significant enough to question whether the rewards of chelation therapy outweigh side effects. At present, the optimal iron chelator for oral formulation is DFP, but DFO is the most versatile chelator overall due to its 1:1 binding ratio and structure which allows for easier chemical manipulation and modifications as needed for delivery via other routes such as intravenous, IN, or oral to improve bioavailability (IV) or uptake (oral, IN). Oral formulations have the most patient compliance and thus are an attractive route of administration for therapies, yet IN formulations can enter the brain without having to address the issue of crossing the BBB. It is our opinion that DFO shows the most promise in treating NBIA and that future research should be directed toward IN and nanoformulations of the drug.

Iron chelators still require significant development and testing before they are a practical treatment to be given to patients with NBIA. Intranasal dosage forms and nanoformulations show potential regarding chelation therapy, but the dosing limitations compared to parenteral and oral formulations are an obstacle that must be overcome for them to go into widespread use. The most current research on NBIA focuses on the use of deferiprone to treat the disorders, but gene therapy provides another pathway of study that would target the direct cause of NBIA. These therapies could allow for correction of the genetic defect before the onset of physical symptoms, negating the need for iron chelators (if diagnosis came after the accumulation of brain iron, then iron chelation therapies would have relevance to prevent further neuronal death). Gene therapies have been shown to have beneficial effects on neurodegeneration and studies have shown great promise for NBIA, such as the use of AAV vectors and CRISPR-Cas9 genome editing in cases of AD, PD, and other neurodegenerative diseases.

Iron chelation therapy may provide a treatment to NBIA, whereas gene therapy may provide a cure. Gene therapies are becoming more accessible to the public, as seen with the recent approval by the FDA of Casgevy and Lyfgenia for use in patients 12 and older with sickle cell disease.^133^ Though this is a major accomplishment, gene therapy is incredibly expensive and is far from being developed for use against an orphan disease such as NBIA. Though there are still many issues to be discussed and questions to be answered, iron chelation is the most realistic treatment that can currently be applied to NBIA, but in the future we hope to see gene therapy used as well. The pathophysiology of NBIA has not yet been completely discovered, but as more research is conducted on the mechanisms of iron accumulation and its associations with neurodegeneration, developing causal treatments for all types of NBIA becomes more possible.

## References

[ref1] Hogarth P. Neurodegeneration with brain iron accumulation: diagnosis and management. J. Mov Disord 2015, 8 (1), 1–13. 10.14802/jmd.14034. 25614780 PMC4298713

[ref2] Bagwe-Parab S.; Kaur G. Molecular targets and therapeutic interventions for iron induced neurodegeneration. Brain Res. Bull. 2020, 156, 1–9. 10.1016/j.brainresbull.2019.12.011. 31866454

[ref3] Orphanet. Neurodegeneration With Brain Iron Accumulation. 2010.: https://www.orpha.net/consor/cgi-bin/OC_Exp.php?Lng=EN&Expert=385 (accessed 2022 October 16).

[ref4] Nunez M. T.; Chana-Cuevas P. New Perspectives in Iron Chelation Therapy for the Treatment of Neurodegenerative Diseases. Pharmaceuticals (Basel) 2018, 11 (4), 109 10.3390/ph11040109. 30347635 PMC6316457

[ref5] Crichton R. R.; Dexter D.T.; Ward R. J. Metal based neurodegenerative diseases—from molecular mechanisms to therapeutic strategies. Coord. Chem. Rev. 2008, 252 (10–11), 1189 10.1016/j.ccr.2007.10.019.

[ref6] Gupta D. Role of Iron (Fe) in Body. IOSR Journal of Applied Chemistry 2014, 7, 38–46. 10.9790/5736-071123846.

[ref7] Sies H. Oxidative Stress: Concept and Some Practical Aspects. Antioxidants (Basel) 2020, 9 (9), 852 10.3390/antiox9090852. 32927924 PMC7555448

[ref8] Kehrer J. P. The Haber-Weiss reaction and mechanisms of toxicity. Toxicology 2000, 149 (1), 43–50. 10.1016/S0300-483X(00)00231-6. 10963860

[ref9] Thomas C.; Mackey M. M.; Diaz A. A.; Cox D. P. Hydroxyl radical is produced via the Fenton reaction in submitochondrial particles under oxidative stress: implications for diseases associated with iron accumulation. Redox Report 2009, 14 (3), 102–108. 10.1179/135100009X392566. 19490751

[ref10] Joppe K.; Roser A.-E.; Maass F.; Lingor P. The Contribution of Iron to Protein Aggregation Disorders in the Central Nervous System. Frontiers in Neuroscience 2019, 13, 15 10.3389/fnins.2019.00015. 30723395 PMC6350163

[ref11] Crichton R. R.; Wilmet S.; Legssyer R.; Ward R. J. Molecular and cellular mechanisms of iron homeostasis and toxicity in mammalian cells. J. Inorg. Biochem 2002, 91 (1), 9–18. 10.1016/S0162-0134(02)00461-0. 12121757

[ref12] Kakhlon O.; Cabantchik Z. I. The labile iron pool: characterization, measurement, and participation in cellular processes1 1This article is part of a series of reviews on “Iron and Cellular Redox Status.” The full list of papers may be found on the homepage of the journal. Free Radical Biol. Med. 2002, 33 (8), 1037–1046. 10.1016/S0891-5849(02)01006-7. 12374615

[ref13] Koppenol W. H.; Hider R. H. Iron and redox cycling. Do’s and don’ts. Free Radic Biol. Med. 2019, 133, 3–10. 10.1016/j.freeradbiomed.2018.09.022. 30236787

[ref14] Cabantchik Z. I. Labile iron in cells and body fluids: physiology, pathology, and pharmacology. Front Pharmacol 2014, 5, 45 10.3389/fphar.2014.00045. 24659969 PMC3952030

[ref15] Eid R.; Arab N. T. T.; Greenwood M. T. Iron mediated toxicity and programmed cell death: A review and a re-examination of existing paradigms. Biochimica et Biophysica Acta (BBA) - Molecular Cell Research 2017, 1864 (2), 399–430. 10.1016/j.bbamcr.2016.12.002. 27939167

[ref16] Minotti G.; Aust S. D. The role of iron in oxygen radical mediated lipid peroxidation. Chem. Biol. Interact 1989, 71 (1), 1–19. 10.1016/0009-2797(89)90087-2. 2550151

[ref17] Catala A.; Diaz M. Editorial: Impact of Lipid Peroxidation on the Physiology and Pathophysiology of Cell Membranes. Front Physiol 2016, 7, 423 10.3389/fphys.2016.00423. 27713704 PMC5031777

[ref18] Fu Y.; He Y.; Phan K.; Bhatia S.; Pickford R.; Wu P.; Dzamko N.; Halliday G. M.; Kim W. S. Increased unsaturated lipids underlie lipid peroxidation in synucleinopathy brain. Acta Neuropathologica Communications 2022, 10 (1), 165 10.1186/s40478-022-01469-7. 36376990 PMC9664712

[ref19] Andrews N. C. Disorders of iron metabolism. N Engl J. Med. 1999, 341 (26), 1986–1995. 10.1056/NEJM199912233412607. 10607817

[ref20] Cadenas B.; Fita-Torro J.; Bermudez-Cortes M.; Hernandez-Rodriguez I.; Fuster J. L.; Llinares M. E.; Galera A. M.; Romero J. L.; Perez-Montero S.; Tornador C.; Sanchez M. L-Ferritin: One Gene, Five Diseases; from Hereditary Hyperferritinemia to Hypoferritinemia-Report of New Cases. Pharmaceuticals (Basel) 2019, 12 (1), 17 10.3390/ph12010017. 30678075 PMC6469184

[ref21] Waldvogel-Abramowski S.; Waeber G.; Gassner C.; Buser A.; Frey B. M.; Favrat B.; Tissot J. D. Physiology of iron metabolism. Transfus Med. Hemother 2014, 41 (3), 213–221. 10.1159/000362888. 25053935 PMC4086762

[ref22] Zhang D. L.; Ghosh M. C.; Rouault T. A. The physiological functions of iron regulatory proteins in iron homeostasis - an update. Front Pharmacol 2014, 5, 124 10.3389/fphar.2014.00124. 24982634 PMC4056636

[ref23] Yanatori I.; Kishi F. DMT1 and iron transport. Free Radical Biol. Med. 2019, 133, 55–63. 10.1016/j.freeradbiomed.2018.07.020. 30055235

[ref24] Gammella E.; Buratti P.; Cairo G.; Recalcati S. The transferrin receptor: the cellular iron gate. Metallomics 2017, 9 (10), 1367–1375. 10.1039/C7MT00143F. 28671201

[ref25] Rouault T. A. Iron metabolism in the CNS: implications for neurodegenerative diseases. Nat. Rev. Neurosci 2013, 14 (8), 551–564. 10.1038/nrn3453. 23820773

[ref26] MacKenzie E. L.; Iwasaki K.; Tsuji Y. Intracellular iron transport and storage: from molecular mechanisms to health implications. Antioxid Redox Signal 2008, 10 (6), 997–1030. 10.1089/ars.2007.1893. 18327971 PMC2932529

[ref27] Dutt S.; Hamza I.; Bartnikas T. B. Molecular Mechanisms of Iron and Heme Metabolism. Annual Review of Nutrition 2022, 42 (1), 311–335. 10.1146/annurev-nutr-062320-112625. PMC9398995 35508203

[ref28] Cheng R.; Dhorajia V. V.; Kim J.; Kim Y. Mitochondrial iron metabolism and neurodegenerative diseases. NeuroToxicology 2022, 88, 88–101. 10.1016/j.neuro.2021.11.003. 34748789 PMC8748425

[ref29] Tian Y.; Tian Y.; Yuan Z.; Zeng Y.; Wang S.; Fan X.; Yang D.; Yang M. Iron Metabolism in Aging and Age-Related Diseases. International Journal of Molecular Sciences 2022, 23 (7), 3612 10.3390/ijms23073612. 35408967 PMC8998315

[ref30] Mills E.; Dong X.-p.; Wang F.; Xu H. Mechanisms of brain iron transport: insight into neurodegeneration and CNS disorders. FUTURE MEDICINAL CHEMISTRY 2010, 2 (1), 51 10.4155/fmc.09.140. 20161623 PMC2812924

[ref31] Rouault T. A.; Zhang D. L.; Jeong S. Y. Brain iron homeostasis, the choroid plexus, and localization of iron transport proteins. Metab Brain Dis 2009, 24 (4), 673–684. 10.1007/s11011-009-9169-y. 19851851 PMC2788140

[ref32] Todorich B.; Pasquini J. M.; Garcia C. I.; Paez P. M.; Connor J. R. Oligodendrocytes and myelination: the role of iron. Glia 2009, 57 (5), 467–478. 10.1002/glia.20784. 18837051

[ref33] Connor J. R.; Menzies S. L. Relationship of iron to oligondendrocytes and myelination. Glia 1996, 17 (2), 83–93. 10.1002/(SICI)1098-1136(199606)17:2<83::AID-GLIA1>3.0.CO;2-7. 8776576

[ref34] Javed K.; Reddy V.; Lui F. Neuroanatomy, Choroid Plexus. In StatPearls, 2023. 30844183

[ref35] Solár P.; Zamani A.; Kubíčková L.; Dubový P.; Joukal M. Choroid plexus and the blood-cerebrospinal fluid barrier in disease. Fluids and Barriers of the CNS 2020, 17 (1), 35 10.1186/s12987-020-00196-2. 32375819 PMC7201396

[ref36] Moos T.; Rosengren Nielsen T.; Skjorringe T.; Morgan E. H. Iron trafficking inside the brain. J. Neurochem 2007, 103 (5), 1730–1740. 10.1111/j.1471-4159.2007.04976.x. 17953660

[ref37] Bradbury M. W. Transport of iron in the blood-brain-cerebrospinal fluid system. J. Neurochem 1997, 69 (2), 443–454. 10.1046/j.1471-4159.1997.69020443.x. 9231702

[ref38] Wiethoff S.; Bhatia K. P.; Houlden H. Genetics of NBIA Disorders. In Movement Disorder Genetics; Schneider S. A., Brás J. M. T., Eds.; Springer International Publishing, 2015; pp 263–291.

[ref39] Hortnagel K.; Prokisch H.; Meitinger T. An isoform of hPANK2, deficient in pantothenate kinase-associated neurodegeneration, localizes to mitochondria. Hum. Mol. Genet. 2003, 12 (3), 321–327. 10.1093/hmg/ddg026. 12554685

[ref40] Hayflick S. J.; Westaway S. K.; Levinson B.; Zhou B.; Johnson M. A.; Ching K. H.; Gitschier J. Genetic, clinical, and radiographic delineation of Hallervorden-Spatz syndrome. N Engl J. Med. 2003, 348 (1), 33–40. 10.1056/NEJMoa020817. 12510040

[ref41] Dansie L. E.; Reeves S.; Miller K.; Zano S. P.; Frank M.; Pate C.; Wang J.; Jackowski S. Physiological roles of the pantothenate kinases. Biochem. Soc. Trans. 2014, 42 (4), 1033–1036. 10.1042/BST20140096. 25109998 PMC4948118

[ref42] Siudeja K.; Srinivasan B.; Xu L.; Rana A.; de Jong J.; Nollen E. A.; Jackowski S.; Sanford L.; Hayflick S.; Sibon O. C. Impaired Coenzyme A metabolism affects histone and tubulin acetylation in Drosophila and human cell models of pantothenate kinase associated neurodegeneration. EMBO Mol. Med. 2011, 3 (12), 755–766. 10.1002/emmm.201100180. 21998097 PMC3377114

[ref43] Orellana D. I.; Santambrogio P.; Rubio A.; Yekhlef L.; Cancellieri C.; Dusi S.; Giannelli S. G.; Venco P.; Mazzara P. G.; Cozzi A.; et al. Coenzyme A corrects pathological defects in human neurons of PANK2-associated neurodegeneration. EMBO Mol. Med. 2016, 8 (10), 1197–1211. 10.15252/emmm.201606391. 27516453 PMC5048368

[ref44] Gregory A.; Hayflick S. J. Genetics of neurodegeneration with brain iron accumulation. Curr. Neurol Neurosci Rep 2011, 11 (3), 254–261. 10.1007/s11910-011-0181-3. 21286947 PMC5908240

[ref45] McNeill A.; Birchall D.; Hayflick S. J.; Gregory A.; Schenk J. F.; Zimmerman E. A.; Shang H.; Miyajima H.; Chinnery P. F. T2* and FSE MRI distinguishes four subtypes of neurodegeneration with brain iron accumulation. Neurology 2008, 70 (18), 1614–1619. 10.1212/01.wnl.0000310985.40011.d6. 18443312 PMC2706154

[ref46] Sonne J.; Reddy V.; Beato M. R. Neuroanatomy, Substantia Nigra. In StatPearls, 2023. 30725680

[ref47] de Leon A. S.; Das J. M. Neuroanatomy, Dentate Nucleus. In StatPearls, 2023. 32119268

[ref48] Gregory A.; Westaway S. K.; Holm I. E.; Kotzbauer P. T.; Hogarth P.; Sonek S.; Coryell J. C.; Nguyen T. M.; Nardocci N.; Zorzi G.; et al. Neurodegeneration associated with genetic defects in phospholipase A(2). Neurology 2008, 71 (18), 1402–1409. 10.1212/01.wnl.0000327094.67726.28. 18799783 PMC2676964

[ref49] Baburina I.; Jackowski S. Cellular responses to excess phospholipid. J. Biol. Chem. 1999, 274 (14), 9400–9408. 10.1074/jbc.274.14.9400. 10092620

[ref50] Marotta N. P.; Ara J.; Uemura N.; Lougee M. G.; Meymand E. S.; Zhang B.; Petersson E. J.; Trojanowski J. Q.; Lee V. M. Y. Alpha-synuclein from patient Lewy bodies exhibits distinct pathological activity that can be propagated in vitro. Acta Neuropathologica Communications 2021, 9 (1), 188 10.1186/s40478-021-01288-2. 34819159 PMC8611971

[ref51] Yong Y.; Hunter-Chang S.; Stepanova E.; Deppmann C. Axonal spheroids in neurodegeneration. Mol. Cell Neurosci 2021, 117, 103679 10.1016/j.mcn.2021.103679. 34678457 PMC8742877

[ref52] Erskine D.; Attems J. Insights into Lewy body disease from rare neurometabolic disorders. Journal of Neural Transmission 2021, 128 (10), 1567–1575. 10.1007/s00702-021-02355-7. 34056672 PMC8528771

[ref53] Gregory A.; Lotia M.; Jeong S. Y.; Fox R.; Zhen D.; Sanford L.; Hamada J.; Jahic A.; Beetz C.; Freed A.; et al. Autosomal dominant mitochondrial membrane protein-associated neurodegeneration (MPAN). Mol. Genet Genomic Med. 2019, 7 (7), e00736 10.1002/mgg3.736. 31087512 PMC6625130

[ref54] Schulte E. C.; Claussen M. C.; Jochim A.; Haack T.; Hartig M.; Hempel M.; Prokisch H.; Haun-Junger U.; Winkelmann J.; Hemmer B.; et al. Mitochondrial membrane protein associated neurodegenration: a novel variant of neurodegeneration with brain iron accumulation. Mov Disord 2013, 28 (2), 224–227. 10.1002/mds.25256. 23436634

[ref55] Hogarth P.; Gregory A.; Kruer M. C.; Sanford L.; Wagoner W.; Natowicz M. R.; Egel R. T.; Subramony S. H.; Goldman J. G.; Berry-Kravis E.; et al. New NBIA subtype: genetic, clinical, pathologic, and radiographic features of MPAN. Neurology 2013, 80 (3), 268–275. 10.1212/WNL.0b013e31827e07be. 23269600 PMC3589182

[ref56] Gagliardi M.; Annesi G.; Lesca G.; Broussolle E.; Iannello G.; Vaiti V.; Gambardella A.; Quattrone A. C19orf12 gene mutations in patients with neurodegeneration with brain iron accumulation. Parkinsonism Relat Disord 2015, 21 (7), 813–816. 10.1016/j.parkreldis.2015.04.009. 25962551

[ref57] Hartig M.; Prokisch H.; Meitinger T.; Klopstock T. Mitochondrial membrane protein-associated neurodegeneration (MPAN). Int. Rev. Neurobiol 2013, 110, 73–84. 10.1016/B978-0-12-410502-7.00004-1. 24209434

[ref58] Haack T. B.; Hogarth P.; Gregory A.; Prokisch H.; Hayflick S. J. Chapter Four - BPAN: The Only X-Linked Dominant NBIA Disorder. In International Review of Neurobiology; Bhatia K. P., Schneider S. A., Eds.; Academic Press, 2013; Vol. 110, pp 85–90. 10.1016/B978-0-12-410502-7.00005-3 24209435

[ref59] Paudel R.; Li A.; Wiethoff S.; Bandopadhyay R.; Bhatia K.; de Silva R.; Houlden H.; Holton J. L. Neuropathology of Beta-propeller protein associated neurodegeneration (BPAN): a new tauopathy. Acta Neuropathol Commun. 2015, 3, 39 10.1186/s40478-015-0221-3. 26123052 PMC4486689

[ref60] Hayflick S. J.; Kruer M. C.; Gregory A.; Haack T. B.; Kurian M. A.; Houlden H. H.; Anderson J.; Boddaert N.; Sanford L.; Harik S. I.; et al. beta-Propeller protein-associated neurodegeneration: a new X-linked dominant disorder with brain iron accumulation. Brain 2013, 136 (Pt 6), 1708–1717. 10.1093/brain/awt095. 23687123 PMC3673459

[ref61] Glick D.; Barth S.; Macleod K. F. Autophagy: cellular and molecular mechanisms. J. Pathol 2010, 221 (1), 3–12. 10.1002/path.2697. 20225336 PMC2990190

[ref62] Mizushima N.; Levine B.; Cuervo A. M.; Klionsky D. J. Autophagy fights disease through cellular self-digestion. Nature 2008, 451 (7182), 1069–1075. 10.1038/nature06639. 18305538 PMC2670399

[ref63] Verhoeven W. M.; Egger J. I.; Koolen D. A.; Yntema H.; Olgiati S.; Breedveld G. J.; Bonifati V.; van de Warrenburg B. P. Beta-propeller protein-associated neurodegeneration (BPAN), a rare form of NBIA: novel mutations and neuropsychiatric phenotype in three adult patients. Parkinsonism Relat Disord 2014, 20 (3), 332–336. 10.1016/j.parkreldis.2013.11.019. 24368176

[ref64] A Study to Assess Efficacy and Safety of RT001 in Subjects With Infantile Neuroaxonal Dystrophy. ClinicalTrials.gov. https://classic.clinicaltrials.gov/show/NCT03570931 (accessed 2023 September 13).

[ref65] Clinical Curative Effect Evaluation Study of Treatment of Oral Deferiprone Tablets in Aceruloplasminaemia Patients. ClinicalTrials.gov. https://classic.clinicaltrials.gov/show/NCT04184453 (accessed 2023 September 13).

[ref66] Iankova V.; Karin I.; Klopstock T.; Schneider S. A. Emerging Disease-Modifying Therapies in Neurodegeneration With Brain Iron Accumulation (NBIA) Disorders. Front Neurol 2021, 12, 629414 10.3389/fneur.2021.629414. 33935938 PMC8082061

[ref67] Cohen A. R. New advances in iron chelation therapy. Hematology Am. Soc. Hematol Educ Program 2006, 2006, 42–47. 10.1182/asheducation-2006.1.42. 17124038

[ref68] Bellotti D.; Remelli M. Deferoxamine B: A Natural, Excellent and Versatile Metal Chelator. Molecules 2021, 26 (11), 3255 10.3390/molecules26113255. 34071479 PMC8198152

[ref69] Liu Z.; Qiao J.; Nagy T.; Xiong M. P. ROS-triggered degradable iron-chelating nanogels: Safely improving iron elimination in vivo. J. Controlled Release 2018, 283, 84–93. 10.1016/j.jconrel.2018.05.025. PMC6035766 29792889

[ref70] Hamilton J. L.; Kizhakkedathu J. N. Polymeric nanocarriers for the treatment of systemic iron overload. Mol. Cell Ther 2015, 3, 3 10.1186/s40591-015-0039-1. 26056604 PMC4451967

[ref71] Cui S.; Liu Z.; Nagy T.; Agboluaje E. O.; Xiong M. P. Oral Non-absorbable Polymer-Deferoxamine Conjugates for Reducing Dietary Iron Absorption. Mol. Pharmaceutics 2023, 20 (2), 1285–1295. 10.1021/acs.molpharmaceut.2c00938. PMC12914557 36622899

[ref72] Hanson L. R.; Fine J. M.; Renner D. B.; Svitak A. L.; Burns R. B.; Nguyen T. M.; Tuttle N. J.; Marti D. L.; Panter S. S.; Frey W. H. Intranasal delivery of deferoxamine reduces spatial memory loss in APP/PS1 mice. Drug Delivery and Translational Research 2012, 2 (3), 160–168. 10.1007/s13346-011-0050-2. 25786865

[ref73] Hanson L. R.; Roeytenberg A.; Martinez P. M.; Coppes V. G.; Sweet D. C.; Rao R. J.; Marti D. L.; Hoekman J. D.; Matthews R. B.; II W. H. F.; Panter S. S. Intranasal Deferoxamine Provides Increased Brain Exposure and Significant Protection in Rat Ischemic Stroke. Journal of Pharmacology and Experimental Therapeutics 2009, 330 (3), 679–686. 10.1124/jpet.108.149807. 19509317 PMC2729791

[ref74] Febbraro F.; Andersen K. J.; Sanchez-Guajardo V.; Tentillier N.; Romero-Ramos M. Chronic intranasal deferoxamine ameliorates motor defects and pathology in the α-synuclein rAAV Parkinson’s model. Exp. Neurol. 2013, 247, 45–58. 10.1016/j.expneurol.2013.03.017. 23531432

[ref75] Guo C.; Wang T.; Zheng W.; Shan Z.-Y.; Teng W.-P.; Wang Z.-Y. Intranasal deferoxamine reverses iron-induced memory deficits and inhibits amyloidogenic APP processing in a transgenic mouse model of Alzheimer’s disease. Neurobiology of Aging 2013, 34 (2), 562–575. 10.1016/j.neurobiolaging.2012.05.009. 22717236

[ref76] Agrawal M.; Saraf S.; Saraf S.; Antimisiaris S. G.; Chougule M. B.; Shoyele S. A.; Alexander A. Nose-to-brain drug delivery: An update on clinical challenges and progress towards approval of anti-Alzheimer drugs. J. Controlled Release 2018, 281, 139–177. 10.1016/j.jconrel.2018.05.011. 29772289

[ref77] Kontoghiorghes G. J.; Aldouri M. A.; Sheppard L.; Hoffbrand A. V. 1,2-Dimethyl-3-hydroxypyrid-4-one, an orally active chelator for treatment of iron overload. Lancet 1987, 329 (8545), 1294–1295. 10.1016/S0140-6736(87)90545-9. 2884415

[ref78] Diav-Citrin O.; Koren G. ORAL IRON CHELATION WITH DEFERIPRONE. Pediatric Clinics of North America 1997, 44 (1), 235–247. 10.1016/S0031-3955(05)70471-5. 9057792

[ref79] Yang L. P. H.; Keam S. J.; Keating G. M. Deferasirox. Drugs 2007, 67 (15), 2211–2230. 10.2165/00003495-200767150-00007. 17927285

[ref80] Jiang X.; Zhou T.; Bai R.; Xie Y. Hydroxypyridinone-Based Iron Chelators with Broad-Ranging Biological Activities. J. Med. Chem. 2020, 63 (23), 14470–14501. 10.1021/acs.jmedchem.0c01480. 33023291

[ref81] Habgood M. D.; Liu Z. D.; Dehkordi L. S.; Khodr H. H.; Abbott J.; Hider R. C. Investigation into the correlation between the structure of hydroxypyridinones and blood-brain barrier permeability. Biochem. Pharmacol. 1999, 57 (11), 1305–1310. 10.1016/S0006-2952(99)00031-3. 10230774

[ref82] Ward R. J.; Dexter D. T.; Crichton R. R. Neurodegenerative diseases and therapeutic strategies using iron chelators. J. Trace Elem Med. Biol. 2015, 31, 267–273. 10.1016/j.jtemb.2014.12.012. 25716300

[ref83] Forni G. L.; Balocco M.; Cremonesi L.; Abbruzzese G.; Parodi R. C.; Marchese R. Regression of symptoms after selective iron chelation therapy in a case of neurodegeneration with brain iron accumulation. Mov Disord 2008, 23 (6), 904–907. 10.1002/mds.22002. 18383118

[ref84] Zorzi G.; Zibordi F.; Chiapparini L.; Bertini E.; Russo L.; Piga A.; Longo F.; Garavaglia B.; Aquino D.; Savoiardo M.; et al. Iron-related MRI images in patients with pantothenate kinase-associated neurodegeneration (PKAN) treated with deferiprone: results of a phase II pilot trial. Mov Disord 2011, 26 (9), 1755–1759. 10.1002/mds.23751. 21557313

[ref85] Cossu G.; Abbruzzese G.; Matta G.; Murgia D.; Melis M.; Ricchi V.; Galanello R.; Barella S.; Origa R.; Balocco M.; et al. Efficacy and safety of deferiprone for the treatment of pantothenate kinase-associated neurodegeneration (PKAN) and neurodegeneration with brain iron accumulation (NBIA): results from a four years follow-up. Parkinsonism Relat Disord 2014, 20 (6), 651–654. 10.1016/j.parkreldis.2014.03.002. 24661465

[ref86] Pratini N. R.; Sweeters N.; Vichinsky E.; Neufeld J. A. Treatment of classic pantothenate kinase-associated neurodegeneration with deferiprone and intrathecal baclofen. Am. J. Phys. Med. Rehabil 2013, 92 (8), 728–733. 10.1097/PHM.0b013e318282d209. 23370589 PMC3707939

[ref87] Klopstock T.; Tricta F.; Neumayr L.; Karin I.; Zorzi G.; Fradette C.; Kmiec T.; Buchner B.; Steele H. E.; Horvath R.; et al. Safety and efficacy of deferiprone for pantothenate kinase-associated neurodegeneration: a randomised, double-blind, controlled trial and an open-label extension study. Lancet Neurol 2019, 18 (7), 631–642. 10.1016/S1474-4422(19)30142-5. 31202468

[ref88] Long-term Deferiprone Treatment in Patients With Pantothenate Kinase-Associated Neurodegeneration. ClinicalTrials.gov. https://classic.clinicaltrials.gov/show/NCT02174848 (accessed 2023 September 13).

[ref89] Efficacy and Safety Study of Deferiprone in Patients With Pantothenate Kinase-associated Neurodegeneration (PKAN). ClinicalTrials.gov. https://classic.clinicaltrials.gov/show/NCT01741532 (accessed 2023 September 13).

[ref90] Compassionate Use of Deferiprone in Patients With PKAN. ClinicalTrials.gov. https://classic.clinicaltrials.gov/show/NCT02635841 (accessed 2023 September 13).

[ref91] Basselin M.; Rosa A. O.; Ramadan E.; Cheon Y.; Chang L.; Chen M.; Greenstein D.; Wohltmann M.; Turk J.; Rapoport S. I. Imaging decreased brain docosahexaenoic acid metabolism and signaling in iPLA(2)beta (VIA)-deficient mice. J. Lipid Res. 2010, 51 (11), 3166–3173. 10.1194/jlr.M008334. 20686114 PMC2952557

[ref92] Cheon Y.; Kim H.-W.; Igarashi M.; Modi H. R.; Chang L.; Ma K.; Greenstein D.; Wohltmann M.; Turk J.; Rapoport S. I.; Taha A. Y. Disturbed brain phospholipid and docosahexaenoic acid metabolism in calcium-independent phospholipase A2-VIA (iPLA2β)-knockout mice. Biochimica et Biophysica Acta (BBA) - Molecular and Cell Biology of Lipids 2012, 1821 (9), 1278–1286. 10.1016/j.bbalip.2012.02.003. 22349267 PMC3393806

[ref93] Yavin E.; Brand A.; Green P. Docosahexaenoic acid abundance in the brain: a biodevice to combat oxidative stress. Nutr Neurosci 2002, 5 (3), 149–157. 10.1080/10284150290003159. 12041873

[ref94] Yu N.; Zhu H.; Yang Y.; Tao Y.; Tan F.; Pei Q.; Zhou Y.; Song X.; Tan Q.; Pei H. Combination of Fe/Cu -chelators and docosahexaenoic acid: an exploration for the treatment of colorectal cancer. Oncotarget 2017, 8 (31), 51478–51491. 10.18632/oncotarget.17807. 28881661 PMC5584262

[ref95] Zhong Y.; Ma C. M.; Shahidi F. Antioxidant and antiviral activities of lipophilic epigallocatechin gallate (EGCG) derivatives. J. Funct Foods 2012, 4 (1), 87–93. 10.1016/j.jff.2011.08.003. 32288792 PMC7105014

[ref96] Mahler A.; Mandel S.; Lorenz M.; Ruegg U.; Wanker E. E.; Boschmann M.; Paul F. Epigallocatechin-3-gallate: a useful, effective and safe clinical approach for targeted prevention and individualised treatment of neurological diseases?. EPMA J. 2013, 4 (1), 5 10.1186/1878-5085-4-5. 23418936 PMC3585739

[ref97] Mandel S. A.; Amit T.; Weinreb O.; Youdim M. B. Understanding the broad-spectrum neuroprotective action profile of green tea polyphenols in aging and neurodegenerative diseases. J. Alzheimers Dis 2011, 25 (2), 187–208. 10.3233/JAD-2011-101803. 21368374

[ref98] Grunblatt E.; Mandel S.; Maor G.; Youdim M. B. Effects of R- and S-apomorphine on MPTP-induced nigro-striatal dopamine neuronal loss. J. Neurochem 2001, 77 (1), 146–156. 10.1046/j.1471-4159.2001.t01-1-00227.x. 11279270

[ref99] Picada J. N.; Roesler R.; Henriques J. A. Genotoxic, neurotoxic and neuroprotective activities of apomorphine and its oxidized derivative 8-oxo-apomorphine. Braz. J. Med. Biol. Res. 2005, 38 (4), 477–486. 10.1590/S0100-879X2005000400001. 15962173

[ref100] Khaliulin I.; Borman J. B.; Chevion M.; Schwalb H. Cardioprotective and Antioxidant Effects of Apomorphine. Free Radical Research 2003, 37 (7), 721–730. 10.1080/1071576031000102150. 12911268

[ref101] Ubeda A.; Montesinos C.; Paya M.; Alcaraz M. J. Iron-reducing and free-radical-scavenging properties of apomorphine and some related benzylisoquinolines. Free Radic Biol. Med. 1993, 15 (2), 159–167. 10.1016/0891-5849(93)90055-Y. 8397141

[ref102] Dairam A.; Fogel R.; Daya S.; Limson J. L. Antioxidant and Iron-Binding Properties of Curcumin, Capsaicin, and S-Allylcysteine Reduce Oxidative Stress in Rat Brain Homogenate. J. Agric. Food Chem. 2008, 56 (9), 3350–3356. 10.1021/jf0734931. 18422331

[ref103] Zhu W.; Xie W.; Pan T.; Xu P.; Fridkin M.; Zheng H.; Jankovic J.; Youdim M. B.; Le W. Prevention and restoration of lactacystin-induced nigrostriatal dopamine neuron degeneration by novel brain-permeable iron chelators. FASEB J. 2007, 21 (14), 3835–3844. 10.1096/fj.07-8386com. 17690154

[ref104] Prachayasittikul V.; Prachayasittikul S.; Ruchirawat S.; Prachayasittikul V. 8-Hydroxyquinolines: a review of their metal chelating properties and medicinal applications. Drug Des Devel Ther 2013, 7, 1157–1178. 10.2147/DDDT.S49763. PMC3793592 24115839

[ref105] Zhu Y.; Chang J.; Tan K.; Huang S. K.; Liu X.; Wang X.; Cao M.; Zhang H.; Li S.; Duan X.; et al. Clioquinol Attenuates Pulmonary Fibrosis through Inactivation of Fibroblasts via Iron Chelation. Am. J. Respir. Cell Mol. Biol. 2021, 65 (2), 189–200. 10.1165/rcmb.2020-0279OC. 33861690

[ref106] Lin G.; Zhu F.; Kanaan N. M.; Asano R.; Shirafuji N.; Sasaki H.; Yamaguchi T.; Enomoto S.; Endo Y.; Ueno A.; et al. Clioquinol Decreases Levels of Phosphorylated, Truncated, and Oligomerized Tau Protein. International Journal of Molecular Sciences 2021, 22 (21), 12063 10.3390/ijms222112063. 34769495 PMC8584684

[ref107] Teil M.; Doudnikoff E.; Thiolat M.-L.; Bohic S.; Bezard E.; Dehay B. The Zinc Ionophore Clioquinol Reduces Parkinson’s Disease Patient-Derived Brain Extracts-Induced Neurodegeneration. Molecular Neurobiology 2022, 59 (10), 6245–6259. 10.1007/s12035-022-02974-5. 35915387

[ref108] Mizutani Y.; Maeda T.; Murate K.; Ito S.; Watanabe H.; Mutoh T. Clioquinol kills astrocyte-derived KT-5 cells by the impairment of the autophagy-lysosome pathway. Arch. Toxicol. 2021, 95 (2), 631–640. 10.1007/s00204-020-02943-8. 33156368

[ref109] Nagle D. G.; Ferreira D.; Zhou Y. D. Epigallocatechin-3-gallate (EGCG): chemical and biomedical perspectives. Phytochemistry 2006, 67 (17), 1849–1855. 10.1016/j.phytochem.2006.06.020. 16876833 PMC2903211

[ref110] Ribaric S. The pharmacological properties and therapeutic use of apomorphine. Molecules 2012, 17 (5), 5289–5309. 10.3390/molecules17055289. 22565480 PMC6268166

[ref111] Xu Z.-C.; Wang X.-B.; Yu W.-Y.; Xie S.-S.; Li S.-Y.; Kong L.-Y. Design, synthesis and biological evaluation of benzylisoquinoline derivatives as multifunctional agents against Alzheimer’s disease. Bioorg. Med. Chem. Lett. 2014, 24 (10), 2368–2373. 10.1016/j.bmcl.2014.03.058. 24726809

[ref112] Moulin S.; Arnaud C.; Bouyon S.; Pepin J. L.; Godin-Ribuot D.; Belaidi E. Curcumin prevents chronic intermittent hypoxia-induced myocardial injury. Ther Adv. Chronic Dis 2020, 11, 204062232092210 10.1177/2040622320922104. PMC7315663 32637058

[ref113] Damiano S.; Longobardi C.; Andretta E.; Prisco F.; Piegari G.; Squillacioti C.; Montagnaro S.; Pagnini F.; Badino P.; Florio S.; Ciarcia R. Antioxidative Effects of Curcumin on the Hepatotoxicity Induced by Ochratoxin A in Rats. Antioxidants (Basel) 2021, 10 (1), 125 10.3390/antiox10010125. 33477286 PMC7830919

[ref114] Galano A.; Martinez A. Capsaicin, a tasty free radical scavenger: mechanism of action and kinetics. J. Phys. Chem. B 2012, 116 (3), 1200–1208. 10.1021/jp211172f. 22188587

[ref115] Colin-Gonzalez A. L.; Santana R. A.; Silva-Islas C. A.; Chanez-Cardenas M. E.; Santamaria A.; Maldonado P. D. The antioxidant mechanisms underlying the aged garlic extract- and S-allylcysteine-induced protection. Oxid Med. Cell Longev 2012, 2012, 907162 10.1155/2012/907162. 22685624 PMC3363007

[ref116] Ng K. T.; Guo D. Y.; Cheng Q.; Geng W.; Ling C. C.; Li C. X.; Liu X. B.; Ma Y. Y.; Lo C. M.; Poon R. T.; et al. A garlic derivative, S-allylcysteine (SAC), suppresses proliferation and metastasis of hepatocellular carcinoma. PLoS One 2012, 7 (2), e31655 10.1371/journal.pone.0031655. 22389672 PMC3289621

[ref117] Numagami Y.; Ohnishi S. T. S-allylcysteine inhibits free radical production, lipid peroxidation and neuronal damage in rat brain ischemia. J. Nutr. 2001, 131 (3s), 1100S–1105S. 10.1093/jn/131.3.1100S. 11238825

[ref118] Shachar D. B.; Kahana N.; Kampel V.; Warshawsky A.; Youdim M. B. Neuroprotection by a novel brain permeable iron chelator, VK-28, against 6-hydroxydopamine lession in rats. Neuropharmacology 2004, 46 (2), 254–263. 10.1016/j.neuropharm.2003.09.005. 14680763

[ref119] Gal S.; Fridkin M.; Amit T.; Zheng H.; Youdim M. B. M30, a novel multifunctional neuroprotective drug with potent iron chelating and brain selective monoamine oxidase-ab inhibitory activity for Parkinson’s disease. J. Neural Transm Suppl 2006, (70), 447–456. 10.1007/978-3-211-45295-0_68. 17017567

[ref120] Spaull R. V. V.; Soo A. K. S.; Hogarth P.; Hayflick S. J.; Kurian M. A. Towards Precision Therapies for Inherited Disorders of Neurodegeneration with Brain Iron Accumulation. Tremor Other Hyperkinet Mov (N Y) 2021, 11, 51 10.5334/tohm.661. 34909266 PMC8641530

[ref121] Hill S. F.; Meisler M. H. Antisense Oligonucleotide Therapy for Neurodevelopmental Disorders. Dev Neurosci 2021, 43 (3–4), 247–252. 10.1159/000517686. 34412058 PMC8440367

[ref122] Ramasamy T.; Ruttala H. B.; Munusamy S.; Chakraborty N.; Kim J. O. Corrigendum to ″Nano drug delivery systems for antisense oligonucleotides (ASO) therapeutics″ [Journal of Controlled Release 352 (2022) 861–878]. J. Controlled Release 2023, 354, 34 10.1016/j.jconrel.2022.12.047. 36397636

[ref123] Boros B. D.; Schoch K. M.; Kreple C. J.; Miller T. M. Antisense Oligonucleotides for the Study and Treatment of ALS. Neurotherapeutics 2022, 19 (4), 1145–1158. 10.1007/s13311-022-01247-2. 35653060 PMC9587169

[ref124] Wang D.; Tai P. W. L.; Gao G. Adeno-associated virus vector as a platform for gene therapy delivery. Nat. Rev. Drug Discov 2019, 18 (5), 358–378. 10.1038/s41573-019-0012-9. 30710128 PMC6927556

[ref125] Abulimiti A.; Lai M. S.; Chang R. C. Applications of adeno-associated virus vector-mediated gene delivery for neurodegenerative diseases and psychiatric diseases: Progress, advances, and challenges. Mech Ageing Dev 2021, 199, 111549 10.1016/j.mad.2021.111549. 34352323

[ref126] Nouri Nojadeh J.; Bildiren Eryilmaz N. S.; Erguder B. I. CRISPR/Cas9 genome editing for neurodegenerative diseases. EXCLI J. 2023, 22, 567–582. 10.17179/excli2023-6155. 37636024 PMC10450213

[ref127] De Plano L. M.; Calabrese G.; Conoci S.; Guglielmino S. P. P.; Oddo S.; Caccamo A. Applications of CRISPR-Cas9 in Alzheimer’s Disease and Related Disorders. Int. J. Mol. Sci. 2022, 23 (15), 8714 10.3390/ijms23158714. 35955847 PMC9368966

[ref128] Colombelli C.; Aoun M.; Tiranti V. Defective lipid metabolism in neurodegeneration with brain iron accumulation (NBIA) syndromes: not only a matter of iron. J. Inherit Metab Dis 2015, 38 (1), 123–136. 10.1007/s10545-014-9770-z. 25300979

[ref129] Farr A. C.; Xiong M. P. Challenges and Opportunities of Deferoxamine Delivery for Treatment of Alzheimer’s Disease, Parkinson’s Disease, and Intracerebral Hemorrhage. Mol. Pharmaceutics 2021, 18 (2), 593–609. 10.1021/acs.molpharmaceut.0c00474. PMC8819678 32926630

[ref130] Devos D.; Labreuche J.; Rascol O.; Corvol J. C.; Duhamel A.; Guyon Delannoy P.; Poewe W.; Compta Y.; Pavese N.; Ruzicka E.; et al. Trial of Deferiprone in Parkinson’s Disease. N Engl J. Med. 2022, 387 (22), 2045–2055. 10.1056/NEJMoa2209254. 36449420

[ref131] Soulieres D.; Mercier-Ross J.; Fradette C.; Rozova A.; Tsang Y. C.; Tricta F. The pharmacokinetic and safety profile of single-dose deferiprone in subjects with sickle cell disease. Ann. Hematol 2022, 101 (3), 533–539. 10.1007/s00277-021-04728-0. 34981144 PMC8810455

[ref132] Elalfy M. S.; Hamdy M.; El-Beshlawy A.; Ebeid F. S. E.; Badr M.; Kanter J.; Inusa B.; Adly A. A. M.; Williams S.; Kilinc Y.; et al. Deferiprone for transfusional iron overload in sickle cell disease and other anemias: open-label study of up to 3 years. Blood Adv. 2023, 7 (4), 611–619. 10.1182/bloodadvances.2021006778. 36018224 PMC9979751

[ref133] U.S. Food and Drug Administration. FDA Approves First Gene Therapies to Treat Patients with Sickle Cell Disease, December 8, 2023. https://www.fda.gov/news-events/press-announcements/fda-approves-first-gene-therapies-treat-patients-sickle-cell-disease (accessed 2024 January 22).

